# The Effect of Bisphasic Calcium Phosphate Block Bone Graft Materials with Polysaccharides on Bone Regeneration

**DOI:** 10.3390/ma10010017

**Published:** 2017-01-01

**Authors:** Hyun-Sang Yoo, Ji-Hyeon Bae, Se-Eun Kim, Eun-Bin Bae, So-Yeun Kim, Kyung-Hee Choi, Keum-Ok Moon, Chang-Mo Jeong, Jung-Bo Huh

**Affiliations:** 1 Department of Prosthodontics, Dental Research Institute, Institute of Translational Dental Sciences, BK21 PLUS Project, School of Dentistry, Pusan National University, Yangsan 50612, Korea; nasis3@naver.com (H.-S.Y.); say0739@daum.net (J.-H.B.); 0228dmqls@hanmail.net (E.-B.B.); cmjeong@pusan.ac.kr (C.-M.J.); 2 Department of Veterinary Surgery, College of Veterinary Medicine, Chonnam National University, Gwangju 61186, Korea; sen0223@gmail.com; 3 Department of Prosthodontics, Pusan National University Hospital, Pusan 49241, Korea; function3@naver.com; 4 Tissue Biotech Institute, Cowellmedi Co., Ltd., Busan 46986, Korea; ckh@cowellmedi.co.kr (K.-H.C.); moonko81@nate.com (K.-O.M.)

**Keywords:** bone regeneration, bone substitutes, composite, biphasic calcium phosphate, carboxymethyl cellulose, crosslinking, hyaluronic acid

## Abstract

In this study, bisphasic calcium phosphate (BCP) and two types of polysaccharide, carboxymethyl cellulose (CMC) and hyaluronic acid (HyA), were used to fabricate composite block bone grafts, and their physical and biological features and performances were compared and evaluated in vitro and in vivo. Specimens of the following were prepared as 6 mm diameter, 2 mm thick discs; BPC mixed with CMC (the BCP/CMC group), BCP mixed with crosslinked CMC (the BCP/c-CMC group) and BCP mixed with HyA (the BCP/HyA group) and a control group (specimens were prepared using particle type BCP). A scanning electron microscope study, a compressive strength analysis, and a cytotoxicity assessment were conducted. Graft materials were implanted in each of four circular defects of 6 mm diameter in calvarial bone in seven rabbits. Animals were sacrificed after four weeks for micro-CT and histomorphometric analyses, and the findings obtained were used to calculate new bone volumes (mm^3^) and area percentages (%). It was found that these two values were significantly higher in the BCP/c-CMC group than in the other three groups (*p* < 0.05). Within the limitations of this study, BCP composite block bone graft material incorporating crosslinked CMC has potential utility when bone augmentation is needed.

## 1. Introduction

A sufficient amount of residual bone is required for a successful outcome for dental implants, and any restoration provided should have a good long-term prognosis. However, hard tissue defects resulting from causes such as infection and trauma often require bone augmentation [1]. Various grafting materials are used for this purpose, such as, autogenic, allogenic, and xenogenic bone and synthetic calcium phosphate bone graft materials [2], and these materials should trigger osteoblast attachment, proliferation, and differentiation by binding to surrounding bone [3]. In addition, they also should degrade appropriately in concert with the speed of bone growth.

Among the bone graft materials, synthetic calcium phosphate bone graft materials, which have excellent biocompatibility, are commonly used as alternatives to autogenous bone or xenograft or allograft materials [4]. These synthetic materials are easily obtained, do not transmit disease and can be manufactured in various forms. Hydroxyapatite (HA) and β-tricalcium phosphate (β-TCP) are representative calcium phosphate graft materials [5,6,7,8]. These materials have drawn interest for bone regeneration because of their structural and chemical similarity with the inorganic component of bone [9]. Although HA is widely used in the dental field as a bone graft material for implant placement due to its excellent biocompatibility and osteoconductivity [5,6], it remains in situ for a long time due to its low in vivo solubility [7]. By comparison, β-TCP quickly dissolves in the body due to its porous structure and low mechanical strength, however this means that the space required for bone regeneration period is often not maintained when β-TCP is used alone [8]. Thus, HA and β-TCP are mixed in various ratios to form biphasic calcium phosphate (BCP) to optimize their advantages [10,11]. It is possible to adjust the degradation rate, mechanical property and the bioactivity of these materials [12].

Commercial forms of particle type synthetic calcium phosphate bone graft materials of various sizes have been marketed. These materials are grafted into defected sites and are covered with a membrane during the guided bone regeneration (GBR) procedure [1,13]. Spaces between particles promote cell invasion and angiogenesis, but also cause mechanical weakening [14]. Furthermore, when the shape of a defect is unfavorable or the size of a defect is large, the augmented site can easily collapse and the particle type graft material is often displaced or lost [14,15,16,17,18]. Therefore, various methods of preventing the escape of particle type bone graft materials have been suggested. Torres et al. [19] tried to prevent this from recipient sites by mixing platelet rich plasma (PRP) with particle type bone grafts, and Dung and Tu used a cap on calvarial defect sites in a rabbit model. However, the results obtained were less than satisfactory [20].

To overcome these problems, studies on the composite materials of organic and inorganic substances have been actively conducted to take advantage of the benefits of block bone and particle type bone graft materials [14,18]. Some of these studies involved introducing organic substances between bone graft particles to prevent particle loss and enhance handling properties. Due to their excellent formability, these materials can be cut or pressed into any shape to help maintain grafts at recipient sites [18,21]. In addition, the introduction of organic substances prevents structural collapse during bone graft degradation process, because their resorption rates and bone cell invasion rates are similar, and, as a result, organic substance absorption harmonizes the bone remodeling processes [21]. Collagen is a representative biodegradable material that is used as an organic scaffold for composite block bone grafts. Such composite block bone grafts are used in various clinical procedures like socket preservation and typical GBR procedures [18,22,23]. However, although they have good handling properties and produce excellent bone augmentation results, they are more expensive than xenogenic bone graft substitutes, and, as a result, several studies have been conducted on the use of degradable polymer graft materials for bone regeneration [24,25,26].

In this study, we used a carboxymethyl cellulose (CMC) and hyaluronic acid (HyA) to prevent particle loss and to enhance the handling properties of bone graft materials. CMC is a polysaccharide used in the food, pharmaceutical, textile, and paper industries. It is biocompatible, biodegradable, cathodic in nature, and promotes calcium phosphate mineralization. Studies on its use in bone regeneration have being actively pursued [27]. On the other hand, HyA is a water-soluble polysaccharide and a type of cathodic glycosaminoglycan, and is widely distributed in all animal tissues. HyA has affinity for calcium phosphate, a major component of extracellular matrix and joints [28,29]. When HyA is added to a particle type bone graft, viscosities are increased, and, thus, graft handling properties improved, and stability of the grafted site can be maintained [30].

CMC and HyA have been confirmed to exhibit in vivo stability, and their uses in the medical field have been extensively studied [31,32]. However, the use of their composites for bone augmentation the dental fields has not been well studied. In this study, we investigated the physical properties of BCP block bone graft materials incorporating CMC or crosslinked CMC or HyA, and compared and evaluated their biological features and performances as bone graft materials in a rabbit calvarial defect model.

## 2. Materials and Methods

### 2.1. Materials

**Figure 1:**
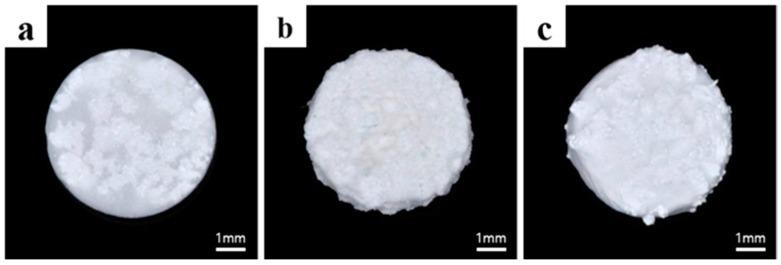
The specimens used in this study: (**a**) bisphasic calcium phosphate/carboxymethyl cellulose (BCP/CMC); (**b**) bisphasic calcium phosphate/cross-linked carboxymethyl cellulose (BCP/c-CMC); and (**c**) bisphasic calcium phosphate/ hyaluronic acid (BCP/HyA).

The BCP (Bio-C, Cowellmedi Co., Ltd., Pusan, Korea) used in this study was a mixture of HA and β-TCP (3:7 ratio; Ca/P ratio 1.55). Materials were prepared as follows. To produce BCP/CMC and BCP/HyA, BCP (0.01 ± 0.002 g) was mixed with CMC (1.5%, Daejung Chem Co., Ltd., Siheung, Korea) or HyA (2.5%, Bioland Co., Ltd., Chunan, Korea) at a ratio of 1:1, and specimens were then freeze-dried on a 96-well plate at −70 °C for 24 h. To prepare BCP containing cross-linked CMC (BCP/c-CMC), 2.5% CMC was mixed with 1% ammonium persulfate (Sigma-Aldrich Corp., St. Louis, MO, USA) and 1% sodium hydrogen sulfite (Sigma-Aldrich Corp.), and then 20% of 2-hydroxyethyl methacrylate monomer (C_6_H_10_O_3_, HEMA, Sigma-Aldrich Corp.) was added to crosslink the CMC, as previously described [33]. The prepared solution was mixed with BCP, reacted in a water bath at 40 °C for 2 h and dried at room temperature for 16 h, and the mixture so obtained was dried in oven at 60 °C for 1 h, and freeze-dried on a 96-well plate at −80 °C for 48 h. Specimens were washed with distilled water 3 times for 10 min on a sonicator (JAC-2010, Kodo Co, Ltd., Hwaseong, Korea), and then freeze-dried on a 96-well plate at −80 °C for 48 h. All specimens were prepared as 6 mm diameter, 2 mm thick block bone discs (Figure 1).

The specimens were divided based on composition into a control group (particle type BCP) and three experimental groups the BCP/CMC, BCP/c-CMC, and BCP/HyA groups.

### 2.2. Physical Characterization

#### 2.2.1. Scanning Electron Microscope Surface Analysis

Specimen surfaces were observed using a scanning electron microscope (SEM, SUPRA 25, Carl Zeiss AG, Oberkochen, Germany) at a magnification of ×500 and ×3000 to assess surface microstructures. The specimens were coated with platinum using a sputter coater (Eiko IB, Tokyo, Japan) and observations were conducted at an accelerating voltage of 10 kV. For surface compositional analyses, the SEM-observed specimens were analyzed by EDX (energy dispersive X-ray spectroscopy; Apollo X, Ametek EDAX, Mahwah, NJ, USA) at an accelerating voltage of 15 kV.

#### 2.2.2. Compressive Strength Analysis

To measure compressive strengths, specimens of BCP/CMC, BCP/c-CMC, and BCP/HyA were prepared of diameter 10 mm and thickness 2 mm (*n* = 5). Loads were applied at 0.5 ± 0.1 mm/min using a universal testing machine (3366, Instron Co., Ltd., Norwood, MA, USA). Obtained load data were divided by cross-section area, and are shown in diagram as a stress (N/cm^2^, log scale) versus distance (μm, linear scale) plot. Maximum stress (N/cm^2^) before fracture was recorded.

#### 2.2.3. In Vitro Cell Test; Assessment of Cytotoxicity

Human MG-63 osteoblast-like cells were seeded into 24-well culture plates containing 0.1 g of specimens per well at a density of 5 × 10^4^ cells/well. Plates were cultured in Dulbecco’s modified eagle’s medium (DMEM, Gibco BRL, Paisley, UK) containing 10% fetal bovine serum (FBS, Gibco BRL), 100 U/mL penicillin (Gibco BRL) for 24 or 72 h at 37 °C in a 5% CO_2_ atmosphere. The effects of specimens on cell proliferation were evaluated using a cell counting Kit-8 (Dojindo, Tokyo, Japan). Experiments were performed five times in in triplicate.

#### 2.2.4. Statistical Analysis

SPSS ver. 21.0 (SPSS, Chicago, IL, USA) was used for the statistical analysis. The significances of differences were determined by One-way analysis of variance (ANOVA). Statistical significance was accepted for *p* values of <0.05.

### 2.3. In Vivo Experiments

#### 2.3.1. Experimental Animals

Seven 12- to 13-week-old male New Zealand white rabbits of average weight 3.4 kg were used in this study. Animals were individually housed in a light- and temperature-controlled environment and provided food and water ad-libitum. Animal selection, animal management, and the surgical procedure were performed in accordance with the standards issued by the Ethics Committee on Animal Experimentation at Chungbuk University (CA-15-13).

#### 2.3.2. Surgical Procedure

**Figure 2:**
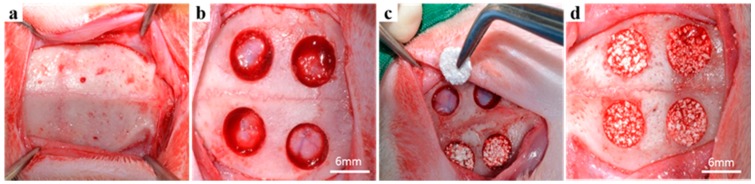
Surgical procedures for the in vivo study: (**a**) parietal bones were exposed by removing periosteum; (**b**) four calvarial defects were formed with a trephine bur (6 mm diameter); (**c**) the four bone grafting materials were randomly placed in defects; and (**d**) all defects were filled with graft materials.

General anesthesia was induced by an intramuscular injection of 0.5 mL tiletamine plus zolazepam (125 mg/mL; Zoletil, Bayer Korea, Seoul, Korea) and 0.5 mL xylazine hydrochloride (10 mg/kg body weight; Rompun, Bayer Korea). The cranium of each animal was shaved and disinfected with povidone-iodine, and the surgical site was injected with 1 mL of 2% lidocaine HCL and 1:100,000 epinephrine (Yu-Han Co., Gunpo, Korea). An about 30 mm incision was made on the skull to expose the parietal bones, and four 6-mm-diameter calvarial defects were produced on each rabbit using a dental-trephine bur as described previously [34,35,36]. The same amounts (0.03 ± 0.002 g) of BCP (Bio-C, Cowellmedi, Pusan, Korea), BCP/CMC, BCP/c-CMC, and BCP/HyA were randomly placed on each calvarial defect (Figure 2). No additional membrane was used. The periosteum was sutured using 4-0 Vicryl^®^ (Johnson & Johnson, New Brunswick, NJ, USA), and skin was sutured using 3-0 silk (Ailee. Co., Ltd., Seoul, Korea).

#### 2.3.3. Postoperative Care and Sacrifice

After surgery, rabbits received 1 mg/kg gentamicin (Kookje, Seoul, Korea) and 0.5 mL/kg Pyrin (Green Cross Veterinary Products, Seoul, Korea) intramuscularly three times daily for 3 days. Animals were allowed to recover for 4 weeks, when they were sacrificed by CO_2_ inhalation. Calvarial defect sites were harvested along with surrounding bone, and harvested specimens were fixed in neutral buffered formalin (Sigma-Aldrich Corp.) for 2 weeks.

#### 2.3.4. Micro-Computed Tomography Analysis

**Figure 3:**
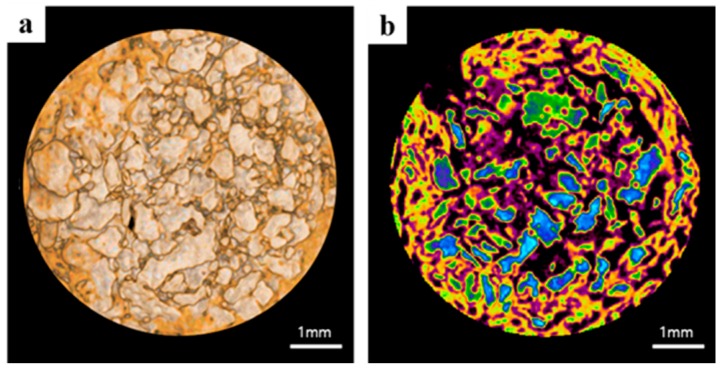
In micro-computed tomography analysis, images of a region of interest: (**a**) reconstructed image; and (**b**) color images of bone graft material and new bone.

After fixation, micro-computed tomography three-dimensional images were obtained to determine new bone volumes in defect areas. Specimens were wrapped in Parafilm M^®^ (Bemis Company, Inc., Neenah, WI, USA) to keep them from drying during scanning and scanned using the following settings; scan energy 130 kV, intensity 60 μA, and a pixel resolution of 7.10 μm using a bromine filter (0.25 mm) (Skyscan-1173, version 1.6, Bruker-CT, Kontich, Belgium). The NRecon reconstruction program (version 1.6.10.1, Bruker-CT, Kontich, Belgium) was performed using the same applied scan and reconstruction parameters for all specimens. New bone volumes (NBV; mm^3^) were calculated within the regions of interest (Figure 3).

#### 2.3.5. Histomorphometric Analysis

**Figure 4:**
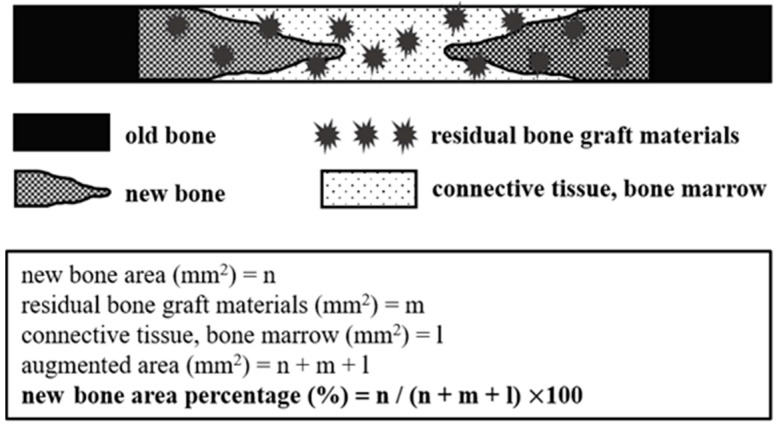
Schematic drawing showing the histometric analysis.

After the micro-CT analysis, specimens were cleaned with distilled water, and calcium was removed using EDTA solution (10%, pH 7.0). After confirming calcium removal, specimens were dehydrated by increasing the ethanol concentration. The alcohol was then removed, and samples were infiltrated with paraffin (PolyFin; Triangle Biomedical Sciences, Durham, NC, USA), paraffin embedded, sectioned longitudinally at 4 µm through each defect center using a microtome (Leica RM2255, Leica Microsystems, Wetzlar, Germany), and mounted on slides. Sections were hematoxylin and eosin (H&E) and Masson’s trichrome stained to visualize newly regenerated bone tissues. The central-most sections from each block were selected for histologic and histometric evaluations. Images of selected slides were captured using an optical microscope connected to a computer (BX51, OLYMPUS, Tokyo, Japan), a charged-coupled device (CCD) camera (SPOT Insight 2Mp scientific CCD digital camera system, DIAGNOSTIC Instruments Inc., Sterling Heights, MI, USA), and an adaptor (U-CMA3, OLYMPUS, Tokyo, Japan). Captured images were analyzed using i-Solution ver. 8.1 (IMT i-Solution, Inc., Coquitlam, BC, Canada). General specimen images were conducted at ×20 and histometric analyses at ×40 and ×100. The histometric analysis was conducted by one professionally trained, blinded investigator. New bone area percentages (%) (defined as defect area occupied by new bone expressed as a percentage of defect area) within defects area were recorded (Figure 4).

#### 2.3.6. Statistical Analysis

Experiment results are presented as means, standard deviations, and medians. Software R (version 3.1.3, R Foundation for Statistical Computing, Vienna, Austria) was used for the statistical analyses. Brunner and Langer nonparametric analysis was to determine the significances of differences [37]. Statistical significance was accepted for *p* values of <0.05.

## 3. Results

### 3.1. Physical Characterization

#### 3.1.1. Scanning Electron Microscope Surface Analysis

**Figure 5:**
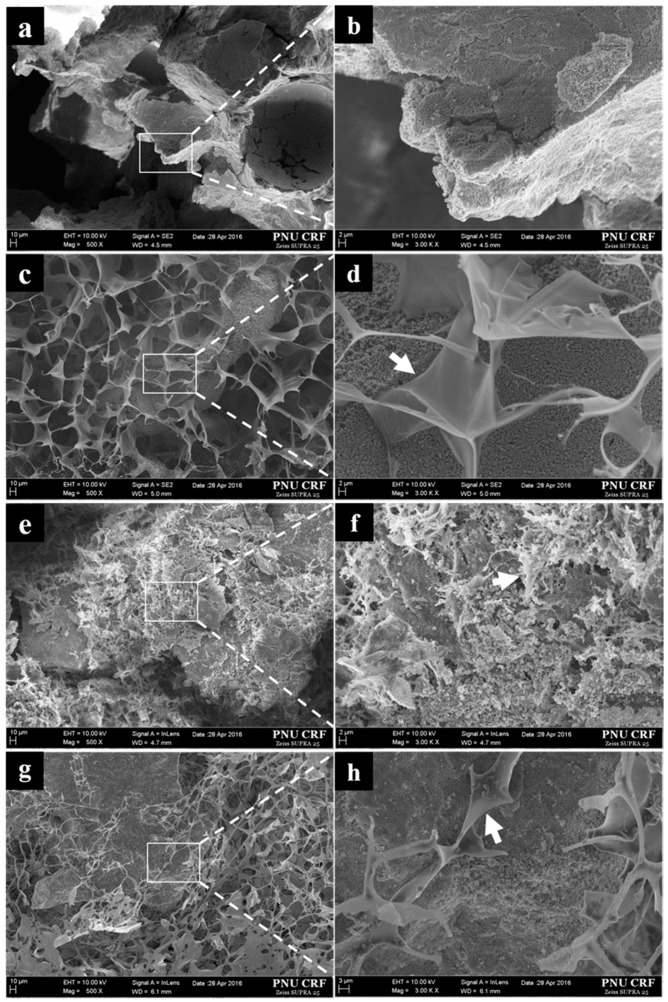
SEM images of bone graft materials. Surfaces of: (**a**,**b**) BCP; (**c**,**d**) BCP/CMC; (**e**,**f**) BCP/c-CMC; and (**g**,**h**) BCP/HyA samples. White arrow indicates polysaccharides (original magnifications: ×500 (**a**,**c**,**e**,**g**); and ×3000 (**b**,**d**,**f**,**h**)).

The surface patterns observed by SEM, and EDX surface compositional analysis results are summarized in Figure 5 and Table 1, respectively. In the BCP/CMC (Figure 5c,d) and BCP/HyA groups (Figure 5g,h), radial type polysaccharides covered the surface of BCP, whereas, in the BCP/c-CMC group, CMC was crosslinked and condensed (Figure 5e,f). EDX showed the presence of Ca and P (major components of the bone graft material) and of C, O, Na, and S (major components of the polysaccharide and the crosslinking agent) (Table 1).

#### 3.1.2. Compressive Strength Analysis

**Figure 6:**
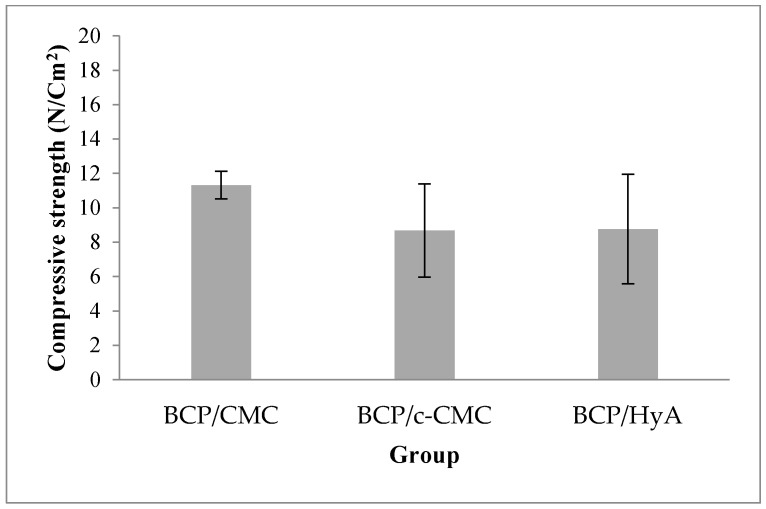
Compressive strengths of the experimental groups. No significant intergroup difference was observed (*p* > 0.05; *n* = 5).

The compressive strength results for each group are shown in Figure 6. The BCP/CMC group produced the highest compressive strengths, followed by the BCP/HyA and BCP/c-CMC groups. However, intergroup differences were not statistically significant (*p* > 0.05).

#### 3.1.3. In Vitro Assessment of Cytotoxicity

**Figure 7:**
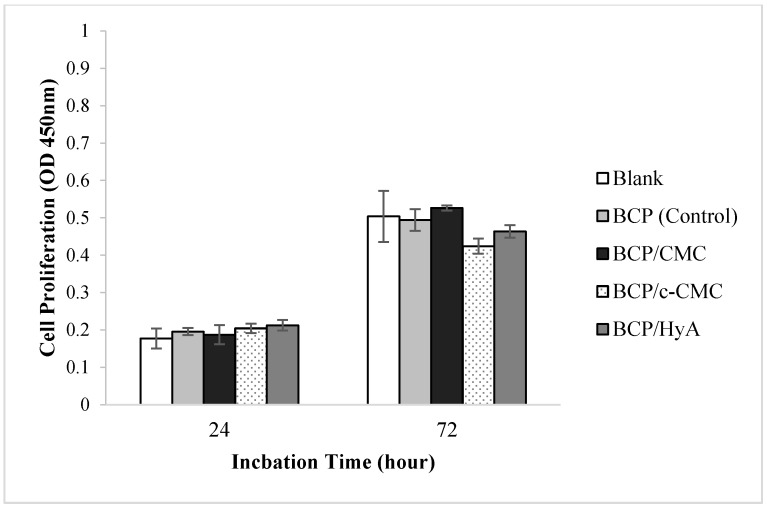
Cytotoxicity of the four graft materials to MG-63 osteoblast-like cells. No evidence of cytotoxicity was observed. Blank, no specimen added (*n* = 5).

Cytotoxicity results for human MG-63 osteoblast-like cells are shown in Figure 7. No evidence of cytotoxicity was observed versus the BCP (control) group.

### 3.2. In Vivo Results

#### 3.2.1. Clinical Findings

All experimental animals survived the surgical procedure, and the 28 defects healed without issue. Furthermore, no infection or inflammation was observed.

#### 3.2.2. Micro-Computed Tomography Findings

**Figure 8:**
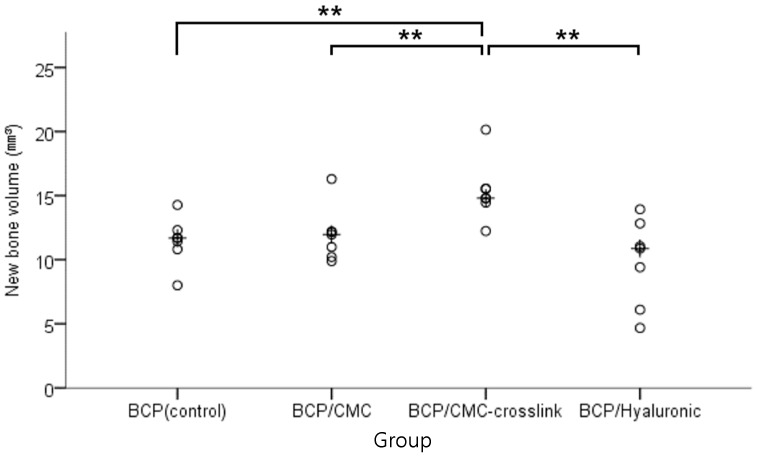
Micro-CT analysis results: scatter plot and median (indicated the cross) representing new bone volumes (mm^3^). The BCP/c-CMC group shows the highest level of new bone production. The symbol “**” indicates statistical significance (*p* < 0.05).

**Figure 9:**
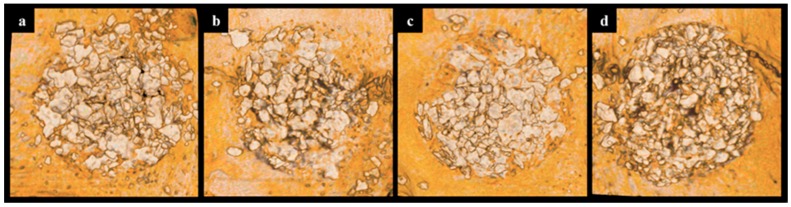
Micro-CT reconstructed images of each group: (**a**) BCP (control) group; (**b**) BCP/CMC group; (**c**) BCP/c-CMC group; and (**d**) BCP/HyA group.

Volumetric measurements are summarized in Table 2 and Figure 8. BCP/c-CMC produced significantly more new bone (mm^3^) than the other three groups at four weeks post surgery (*p* < 0.05), which were not significantly different (*p* > 0.05). Micro-CT images revealed bone graft materials in the BCP/c-CMC group had stabilized on defects and the presence of new bone regeneration, whereas in the other groups graft materials had disintegrated and scattered (Figure 9).

#### 3.2.3. Histologic Findings

**Figure 10:**
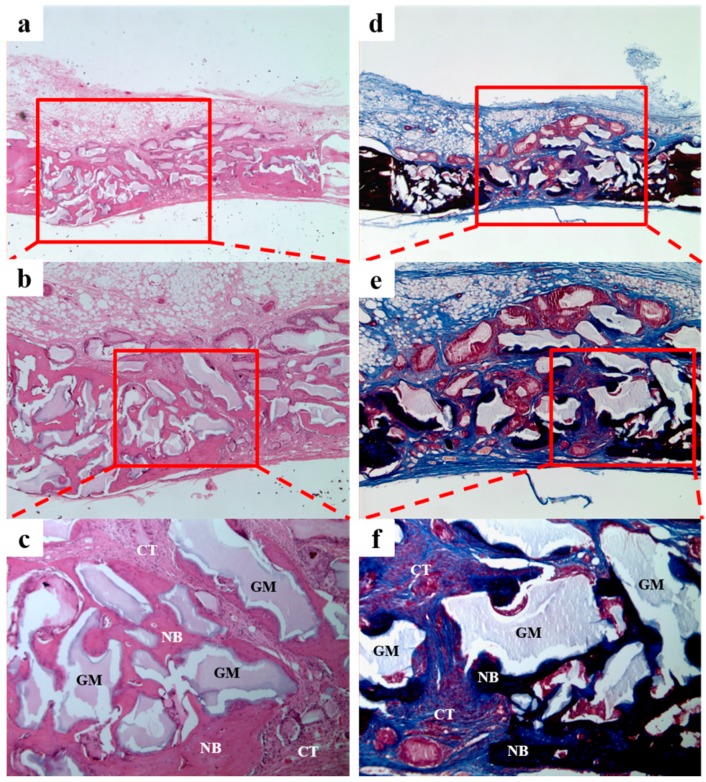
Histological sections of the BCP group at 4 weeks after surgery. A small amount of new bone formation and fibrous connective tissue were observed. NB: new bone; GM: grafted material; CT: connective tissue. Hematoxylin and eosin (H&E) stain results (**a**–**c**); and Masson’s trichrome staining results (**d**–**f**) (original magnifications: ×20 (**a**,**d**); ×40 (**b**,**e**); and ×100 (**c**,**f**)).

In the BCP group (Figure 10), we observed new bone formation derived from existing old bone and around grafted materials. No tissue inflammation was observed.

**Figure 11:**
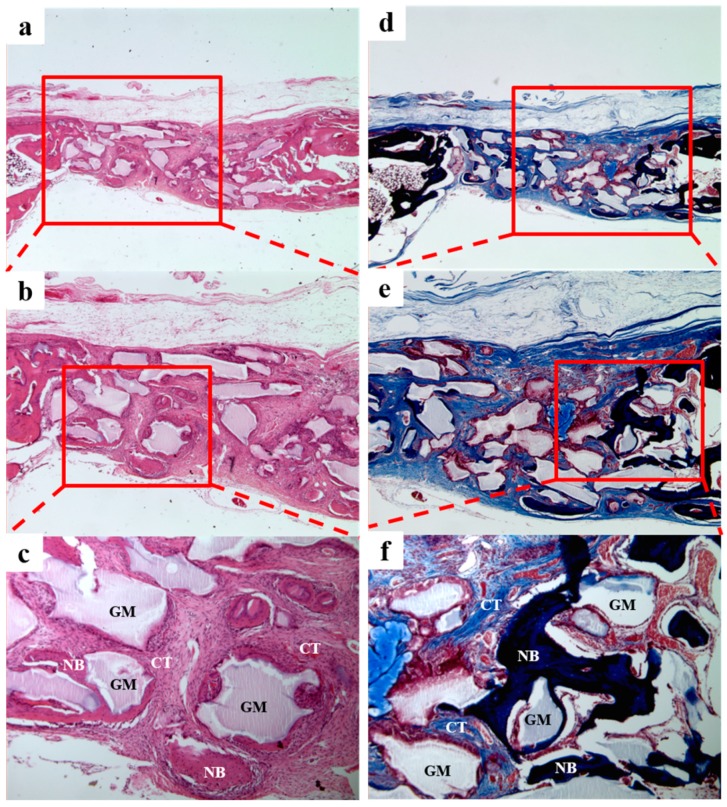
Histological sections of the BCP/CMC group at 4 weeks after surgery. A small amount of new bone formation and a substantial amount of fibrous connective tissue were observed. NB, new bone; GM, grafted material; CT, connective tissue. H&E stain results (**a**–**c**); and Masson’s trichrome stain results (**d**–**f**) (original magnifications: ×20 (**a**,**d**); ×40 (**b**,**e**); and ×100 (**c**,**f**)).

In the BCP/CMC group (Figure 11), we observed a small amount of new bone formation derived from existing bone and around the grafted materials. The grafted material was evenly distributed, and large amounts of fibrous tissues without inflammation were observed throughout defect sites.

**Figure 12:**
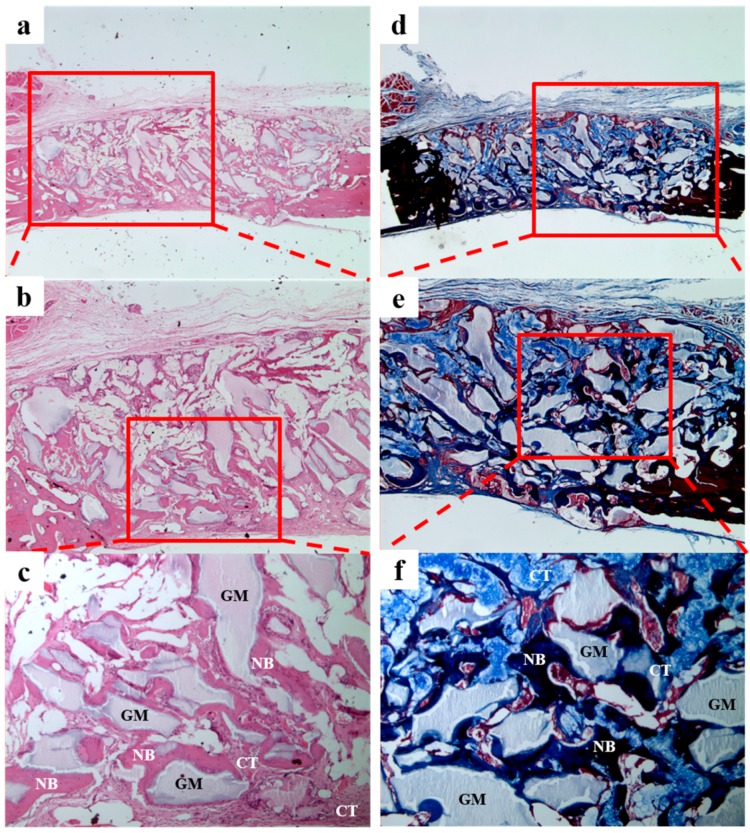
Histological sections of the BCP/c-CMC group at four weeks after surgery. A great amount of new bone formation and a small amount of fibrous connective tissue were observed. NB, new bone; GM, grafted material; CT, connective tissue. H&E stain results (**a**–**c**); and Masson’s trichrome stain results (**d**–**f**) (original magnification: ×20 (**a**,**d**); ×40 (**b**,**e**); and ×100 (**c**,**f**)).

In the BCP/c-CMC group (Figure 12), we observed a large amount of new bone formation derived from existing old bone and grafted material. The boundary of the periosteum was clearly observed, and the bone graft material well occupied defects. No tissue inflammation was observed.

**Figure 13:**
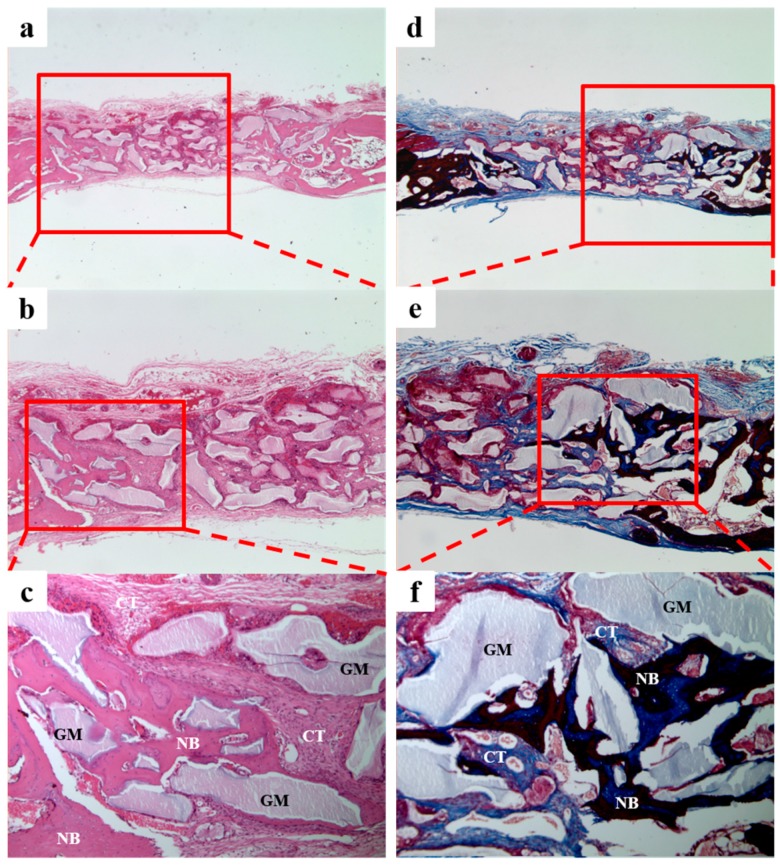
Histological sections of the BCP/HyA group at 4 weeks after surgery. A small amount of new bone formation and fibrous connective tissue were observed. NB, new bone; GM, grafted material; CT, connective tissue. H&E stain results (**a**–**c**); and Masson’s trichrome stain results (**d**–**f**) (original magnification: ×20 (**a**,**d**); ×40 (**b**,**e**); and ×100 (**c**,**f**)).

In the BCP/HyA group (Figure 13), we observed new bone formation derived from existing bone and around grafted material. As was observed in the BCP/CMC group, large amounts of fibrous tissues without inflammation were observed throughout defect sites.

#### 3.2.4. Histometric Findings

**Figure 14:**
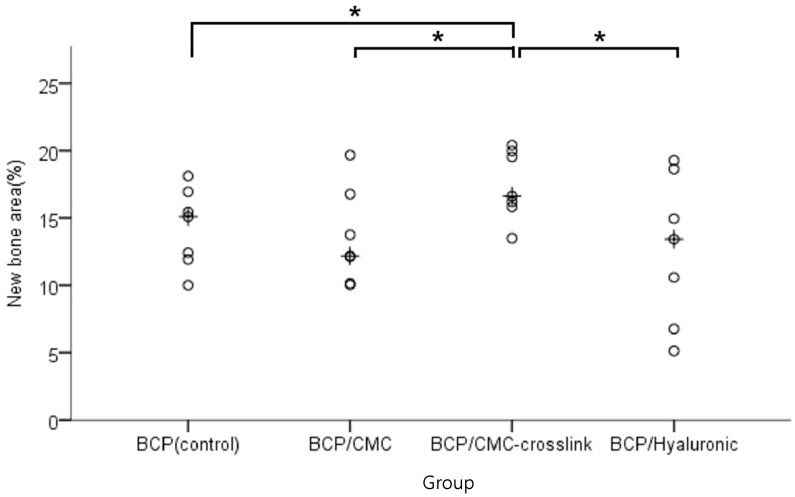
Scatter plot and median (indicated by the cross) of new bone areas (%). The BCP/c-CMC group exhibited more new bone area than the other three groups. The symbol “*” indicates statistical significance (*p* < 0.05).

Histometric measurements are summarized in Table 3 and Figure 14. The BCP/c-CMC group exhibited a significantly higher new bone area percentage (%) (*p* < 0.05). No significant difference was observed between the other three groups (*p* > 0.05).

## 4. Discussion

Many methods can be used to improve the properties of polymers [38,39,40], but crosslinking is the most popular method of improving material properties. For example, as compared with native collagen, crosslinked collagen is resorbed more slowly in vivo and acts a barrier membrane to maintain long-term functional stability [40,41]. Crosslinking also makes it possible incorporate polysaccharides to improve properties [42]. In the case of CMC, the crosslinked block bone was compared with non-crosslinked block bone a to examine differences in physical properties and new bone formation ability. In the present study, crosslinking was conducted using the method previously described for chitosan, which has a chemical structure is similar to that of CMC [33]. We also considered crosslinking HyA for comparison purposes using glutaraldehyde or divinyl sulfone, but it because the use of glutaraldehyde might resulted in toxic residuals and divinyl sulfone is not free of safety concerns [43], we did not include crosslinked HyA in the study.

According to micro-CT results, the BCP/c-CMC group showed about 15 mm^3^ of new bone formation per defect, whereas the other three groups achieved 9–11 mm^3^. Furthermore, micro-CT showed bone grafts in the BCP, BCP/CMC, and BCP/HyA groups had disintegrated and scattered, while in the BCP/c-CMC group, bone grafts were intact and well stabilized in defect sites, which was attributed to crosslinking. However, no significant differences in compressive strength were observed between specimens. These observations concur with those of a previous on the use of crosslinked collagen membranes, which took longer to degrade than corresponding non-crosslinked membranes [41].

In the BCP/CMC and BCP/HyA groups, no difference in new bone formation was observed when particle type BCP was grafted. Because the calvarial defects made were closed, that is, surrounded by a bony wall, dura mater, and periosteum graft materials could not escape as is observed for typical one- or two-wall defects. Thus, the differences among the groups may be significant in the clinical dehiscence defects which cannot maintain the graft materials easily, further study designs may be necessary in the future for confirmation.

Our histometric analysis results of tissue specimens showed the same tendencies as micro-CT results. In the BCP/c-CMC group, new bones accounted for about 17% of defect area, while in the other groups new bones levels ranged from 12% to 14%, which was a significant difference. In tissue specimens, the BCP/c-CMC group showed well maintained graft volumes at defect sites, whereas the graft volumes were reduced in the other groups, particularly in the center areas of defect sites. In addition to maintaining graft volume, it would appear the presence CMC promoted bone metabolism, and that this resulted in the formation of thicker, new bone around grafts in the BCP/c-CMC group than was clearly observed in the other experimental groups.

In histometric analysis of the BCP/CMC and BCP/HyA groups, no significant differences in new bone formation were observed due to the use of particle type BCP (Figure 10b,e). However, the BCP/CMC (Figure 11b,e), BCP/c-CMC (Figure 12b,e), and BCP/HyA (Figure 13b,e) groups showed an obvious boundary between graft and periosteum, whereas in the BCP group the boundary was rough. During GBR (guided bone regeneration) procedures, a membrane is required to prevent soft tissue invasion, but no invasion was observed despite the lack of a membrane in the BCP/CMC, BCP/c-CMC, or BCP/HyA groups, which potentially could simplify the GBR procedure. Zecha et al. reported [44] the application of an absorptive membrane superior to the graft did not result in additional bone formation when bone augmentation was performed using HA/collagen composite block bone. Rothamel et al. [45] found no difference in the bone augmentation results according to the presence/absence of a membrane in allogeneic block bone grafts in a dog model.

The 6 mm diameter defect made in rabbit calvarial bone in the present study was smaller than the 10 mm critical defect size used to evaluate re-ossification in rabbits [46]. However, in the present study, control defects were filled with grafts like the experimental groups. Therefore, there were no limitations in the comparison of new bone formation ability of the graft materials. If a specimen is placed on dura mater, about 10 mmHg intracranial pressure is introduced [47]. This pressure can be assumed to be typical of the pressure applied to a graft by surrounding tissues during GBR. The calvarial bone is mainly composed of cortical bones with relatively smaller bone marrow cells. Thus, graft stability during new bone formation is more critical than in other cell rich areas, which is in-line with the objectives of the present study.

The matrices of the commercially available organic substances used in composite block bone grafts usually consist of collagen. Collagen maintains inorganic particles and blood clots, and has excellent formability, which allows it to be shaped to fit defects. These properties and other make collagen the most useful material for preparing composite materials [18,48]; furthermore, its safety has been well proven in clinical practice. However, no material has ideal properties for bone grafts, and, thus, efforts to identify better new materials are ongoing. In the present study, the BCP/HyA and BCP/CMC groups showed the same ability to promote new bone formation as the BCP control group, and no soft tissue invasion was observed even without a barrier membrane. The BCP/c-CMC group showed most new bone formation, which suggests is has potential use as composite block bone graft material.

## 5. Conclusions

The micro-CT and histomorphometric analysis showed the BCP/c-CMC group promoted most new bone formation and higher new bone area percentages (%). No significant difference was observed between the other three groups in this respect. The present study shows, BCP/crosslinked CMC composite block bone graft material has potential utility as a means of bone augmentation.

## Figures and Tables

**Table 1:** Energy dispersive X-ray spectroscopy (EDX) determined chemical compositions (wt %) in the four study groups.

Elements	Chemical Compositions (wt %)			
	BCP (Control)	BCP/CMC	BCP/c-CMC	BCP/HyA
C	4.2	25.5	30.4	26.5
O	35.7	35.1	36.2	36.3
Na	-	4.3	5.6	-
P	19.4	11.5	8.6	13.6
S	-	-	6.6	-
Ca	40.9	23.5	12.7	24.0

**Table 2:** New bone volumes within regions of interest (*n* = 7; mm^3^).

Group	Mean ± SD	Median
BCP (control)	11.45 ± 1.87	11.69 ^c^
BCP/CMC	11.95 ± 2.13	11.96 ^c^
BCP/c-CMC	15.35 ± 2.39	14.80 ^a,b,d^
BCP/HyA	9.83 ± 3.39	10.87 ^c^
*p* value	<0.05	

^a^ Significantly different versus the BCP (control) group (*p <* 0.05); ^b^ Significantly different versus the BCP/CMC group (*p <* 0.05); ^c^ Significantly different versus the BCP/c-CMC group (*p <* 0.05); ^d^ Significantly different versus the BCP/HyA group (*p <* 0.05).

**Table 3:** New bone area percentage within the region of interest (*n* = 7; %).

Group	Mean ± SD	Median
BCP (control)	14.27 ± 2.92	15.09 ^c^
BCP/CMC	13.52 ± 3.56	12.17 ^c^
BCP/c-CMC	17.43 ± 2.59	16.61 ^a,b,d^
BCP/HyA	12.68 ± 5.49	13.42 ^c^
*p* value	0.033	

^a^ Significantly different versus the BCP(control) group (*p <* 0.05); ^b^ Significantly different versus the BCP/CMC group (*p <* 0.05); ^c^ Significantly different versus the BCP/c-CMC group (*p <* 0.05); ^d^ Significantly different versus the BCP/HyA group (*p <* 0.05).
